# MULTIDIMENSIONAL SLEEP HEALTH IN CARDIOMETABOLIC AND COGNITIVE AGING

**DOI:** 10.1093/geroni/igae098.0848

**Published:** 2024-12-31

**Authors:** Orfeu Buxton

**Affiliations:** Pennsylvania State University, University Park, Pennsylvania, United States

## Abstract

Multi-dimensional sleep health perspectives provide a holistic picture of the various independent and interacting facets of sleep health essential for health and wellbeing. This is key because characterizing sleep extends beyond simple measures such as the amount of time spent asleep and encompasses broad yet interrelated concepts. Taking a life course perspective, this presentation will provide theoretical background and empirical evidence for a 6-component multi-dimensional sleep health model using objective (wrist actigraphy) measures. This will, specifically, be examined in two large cohort studies of well-phenotyped participants. First, using data from the Work, Family and Health Study, evidence will be presented for the relationship of work-related stress, sleep health, and biomarker-based cardiometabolic risk in mid-life and older adults. Second, using data from the Einstein Aging Study, empirical evidence will be presented for the presence of 6-facet multi-dimensional sleep health construct using factor analysis, and its relationship with cognition and mild cognitive impairment. A more comprehensive approach incorporating sleep disorders into a multi-dimensional sleep health framework will also be discussed.

